# Field trials to evaluate the effects of transgenic *cry1Ie* maize on the community characteristics of arthropod natural enemies

**DOI:** 10.1038/srep22102

**Published:** 2016-02-26

**Authors:** Jingfei Guo, Kanglai He, Richard L. Hellmich, Shuxiong Bai, Tiantao Zhang, Yunjun Liu, Tofael Ahmed, Zhenying Wang

**Affiliations:** 1 State Key Laboratory for Biology of Plant Diseases and Insect Pests, MOA – CABI Joint Laboratory for Bio-safety, Institute of Plant Protection, Chinese Academy of Agricultural Sciences, Beijing 100193, China; 2 USDA-ARS, Corn Insects and Crop Genetics Research Unit, Ames, IA 50011, USA; 3 Institute of Crop Sciences, Chinese Academy of Agricultural Sciences, Beijing 100081, China; 4 Entomology Division, Bangladesh Sugarcane Research Institute, Ishurdi, Pabna, Bangladesh

## Abstract

Possible non-target effect of transgenic *cry1Ie* maize exerts on natural enemy community biodiversity in the field is unresolved. In the present study, a 2-yr comparison of transgenic *cry1Ie* maize (Event IE09S034, Bt maize) and its near isoline (Zong 31, non-Bt maize) on natural enemy community biodiversity were compared with whole plant inspections, pitfall traps and suction sampler. Natural enemy diversity indices (Shannon-Wiener’, Simpson’s and Pielou’s index) and abundance suggested there were no significant differences between the two types of maize. The only exceptions were the Pielou’s index for whole plant inspections in 2013 and abundance for pitfall traps in 2012, which were significantly higher in Bt maize than those of non-Bt maize. The main species of natural enemies were identical in Bt and non-Bt maize plots for each method and the three methods combined. For whole plant inspections, Bt maize had no time-dependent effect on the entire arthropod natural enemy community, and also no effect on community dissimilarities between Bt and non-Bt maize plots. These results suggested that despite the presence of a relatively minor difference in natural enemy communities between Bt and non-Bt maize, transgenic *cry1Ie* maize had little, if any, effect on natural enemy community biodiversity.

Populations of corn borers, such as European corn borer (ECB), *Ostrinia nubilalis* (Hübner) and Asian corn borer (ACB), *O. furnacalis* (Guenée) (Lepidoptera: Crambidae), can be drastically reduced by planting transgenic insect-resistant maize, *Zea mays* L.^1^,^2^. Suppression of Lepidopteran pests provides benefits for human health and the environment by reducing use of conventional insecticides^3^. In 2014, the total cultivation of genetically modified (GM) maize was more than 55.2 million hectares, which globally is 30% of the 184 million hectares of maize planted^4^. Before new GM maize varieties are commercialized, environmental risk assessments (ERA) are conducted to determine potential negative effects on non-target species, especially beneficial species that occur in agricultural ecosystems^5^,^6^,^7^.

The most cultivated GM maize lines are those that produce one or more *Bacillus thuringiensis* (Bt) Cry proteins. Currently these Cry proteins kill a narrow range of lepidopteran pests such as ECB and ACB^8^ or coleopteran pests such as corn rootworm, *Diabrotica* spp^9^. Maize Bt proteins are produced throughout the growing season, so natural enemies may be exposed to these proteins directly by feeding on Bt tissues (e.g., pollen) or indirectly by consuming prey that have fed on Bt maize^10^. Widespread adoption of Bt maize has raised concerns by some scientists about potential impacts of Bt maize on arthropod community biodiversity, especially parasitoid, predator, decomposers and pollinators^5^.

When predators prey on herbivores (target or non-target pests) or parasitoids develop on host arthropods, Bt proteins could be transmitted to a higher trophic level, thereby these natural enemies could be exposed to Bt proteins^11^. Previous studies confirm that indeed some predators are exposed to Bt proteins in a plant-herbivore-predator (tritrophic) system^12^.

There are many examples of Bt proteins exposure in bitrophic and tritrophic arthropod systems. For example, bitrophic exposure of Asian ladybird beetle, *Harmonia axyridis* (Coleoptera: Coccinellidae) to Cry3A proteins occurs when they directly consume Bt potato, *Solanum tuberosum* L.^13^. Likewise, some predatory natural enemies, such as the ladybird beetle, *Propylea japonica* (Thunberg) (Coleoptera: Coccinellidae), directly forage on plant pollen, as a complementary food source^14^. Tritrophic exposure of Cry proteins occurs in the following systems: (Cry crop: predator, prey) Cry1Ab maize: rove beetle, *Atheta coriaria* (Kraatz) (Coleoptera: Staphylinidae) larvae and adults, two-spotted spider mite, *Tetranychus urtica*e (Koch) (Acari: Tetranychidae)^15^; Cry1Ab maize: fall armyworm, *Spodoptera frugiperda* (J.E. Smith), *H. axyridis* larvae and adults^16^; Cry1Ab rice, *Oryza sativa* L.: wolf spider, *Pirata subpiraticus* (Bösenberg et Strand) (Araneae: Lycosidae), leaffolder, *Cnaphalocrocis medinalis* (Guenée) (Lepidoptera: Pyralidae)^17^; Cry1Ac broccoli, *Brassica oleracea* L. var. *italica* Plenck,: green lacewings, *Chrysoperla rufilabris* (Burmeister) (Neuroptera: Chrysopidae), cabbage looper, *Trichoplusia ni* (Hübner) (Lepidoptera: Noctuidae); Cry1Ac/Cry2Ab cotton: green lacewings, fall armyworm; and Cry1F maize: green lacewings, fall armyworm^18^. Bioaccumulation of Cry proteins may alter the biology and behavior of natural enemies^19^, and could reduce the species richness and abundance. Natural enemies as biocontrol organisms play a fundamental role in providing ecosystem services and maintaining ecosystem function^20^.

A number of field trials have been conducted to assess the potential negative impacts of Cry1Ab, Cry1Ac and Cry3Bb1 maize on natural enemies. Results suggested Cry1Ab maize had no significant effect on population dynamics of ladybird beetles (Coleoptera: Coccinellidae)^21^, and also was compatible with other natural enemies from the families Anthocoridae, Coccinellidae, and Araneae^22^. In addition, a 2-yr field study suggested Bt maize varieties expressing insecticidal proteins of Cry34Ab1, Cry35Ab1, Cry1F also had no adverse effects on arthropod food web properties^23^. Laboratory studies feeding *Trichogramma ostriniae* (Chen & Pang) (Hymenoptera: Trichogrammatidae) with Cry1Ab maize pollen suggested Cry1Ab maize had no adverse effects on longevity and fecundity of *T. ostriniae*^24^. Likewise, Cry3Bb1 maize had no acute or chronic fitness effects on *Coleomegilla maculata* (DeGeer) (Coleoptera: Coccinellidae) that fed on aphid^25^. However, possible non-target effects on natural enemies also must be assessed for new types of Bt maize^26^. Such an assessment includes numerous factors, such as expression rates of the Cry protein in different plant tissues over the growing season, ingestability and susceptibility of natural enemies to the Cry proteins^12^, transfer probabilities of Cry proteins to higher trophic levels^17^, feeding ecology of both herbivores and natural enemies^27^, and influence of Cry proteins on predator behavior^28^.

The c*ry1Ie* gene was first successfully identified from *B. thuringiensis* isolate Btc007 in Institute of Plant Protection, Chinese Academy of Agricultural Sciences^29^. *Cry1Ie* gene encodes insecticidal proteins toxic to ACB and cotton bollworm, *Helicoverpa armigera* (Hübner) (Lepidoptera: Noctuidae)^30^. This gene shows no cross resistance with Cry1Ab, Cry1Ac, Cry1Ah and Cry1F insecticidal proteins^31^,^32^,^33^,^34^. A study on environmental risk assessment of transgenic *cry1Ie* gene maize on arthropod biodiversity was conducted^35^, but did not include a focused analysis of the natural enemy’s data. Because of the importance of natural enemies to the maize ecosystem, the present study will reevaluate the natural enemy data from that study in more detail.

The aim is to compare arthropod natural enemies in transgenic *cry1Ie* maize plots with those in near isoline Zong 31 maize plots with emphases on (i) species diversity and abundance, (ii) time-dependent effects of Bt maize on community composition, (iii) similarities of community structures between Bt and non-Bt maize and their response to maize type and sampling time. With this purpose, the abundance and diversity of natural enemies from the two-year field study were evaluated. The three multivariate techniques, redundancy analysis (RDA), principal response curve (PRC), and nonmetric multidimensional scaling (nMDS) were used to assess the community data of natural enemies in Bt and non-Bt maize plots.

## Results

### Natural enemies in Bt and non-Bt maize plots

**Figure 1:**
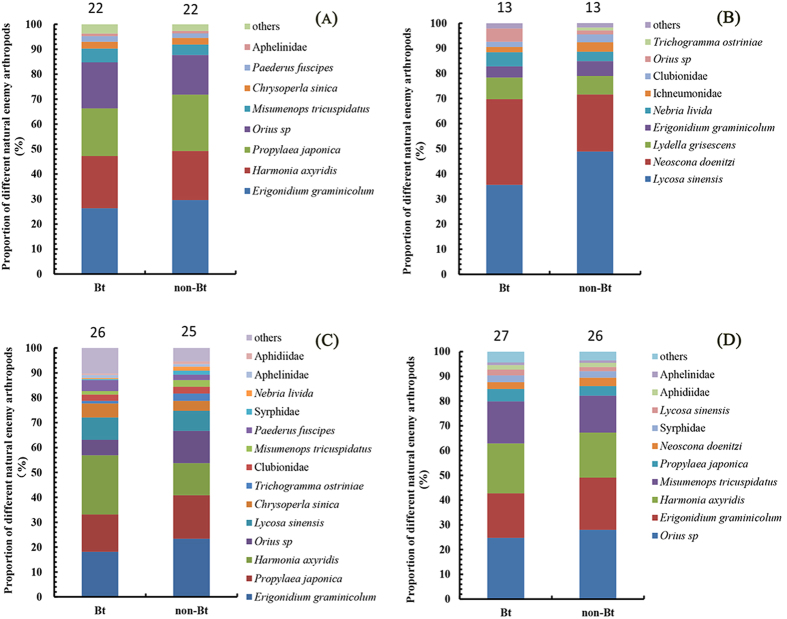
Proportional representation of natural enemies found in Bt and non-Bt maize plots in 2012 and 2013 by whole plant inspections (**A**), pitfall traps (**B**), suction sampler (**C**) and three methods combined (**D**). The Y-axis shows the percentage of each taxa and the X-axis shows the maize type. This diagram illustrates dominant natural enemies (proportion > 1%) and the total proportion of taxa with percentages below 1% (others). Numbers above the columns show the total numbers of taxa collected with each method and three methods combined.

Over the two year study for whole plant inspections, there were 6795 natural enemies (22 species/families) documented in non-Bt maize plots and 6527 (22 species/families) documented in Bt maize plots (Table S1). The most abundant natural enemies in Bt and non-Bt maize plots were similar, including *Erigonidium graminicolum* (Sundevall) (Araneida: Micryphantidae). *P. japonica*, *H. axyridis*, *Orius* sp. and *Misumenops tricuspidatus* (Fabricius) (Araneae: Thomisidae). These taxa comprised more than 90% of all observed natural enemies (Fig. 1A, Table S1). In pitfall traps, 423 individuals (13 species/families) were collected in non-Bt maize plots, and 337 individuals (13 species/families) were collected in Bt maize plots, fewer than those from whole plant inspections. Taxa occurrences also were similar in Bt and non-Bt maize plots over two years, with the exception that *P. japonica* was observed only once in Bt maize plots, and *T. ostriniae* was observed only five times in non-Bt maize plots. *Lycosa sinensis* (Schenkel) (Araneae: Lycosidae) represented nearly half of the natural enemy community in non-Bt maize plots and more than one third in Bt maize plots (Fig. 1B). For suction samplers, 372 individuals (25 species/families) and 480 individuals (26 species/families) were collected in non-Bt and Bt maize plots. The most abundant species in non-Bt and Bt maize plots were the same. The percentages for those species were *E. graminicolum* (23.39%), *H. axyridis* (12.90%), *P. japonica* (17.47%) and *Orius* sp. (12.90%) in non-Bt maize plots, and *E. graminicolum* (18.13%), *P. japonica* (15.00%), *H. axyridis* (23.75%), and *Orius* sp. (6.25%) in Bt maize plots (Fig. 1C, Table S1). Altogether in the three methods over two years, 7590 individuals (26 identified species/families) were found in non-Bt maize plots and 7344 individuals (27 identified species/families) were found in Bt maize plots. Only one *Coccinella septempunctata* L. (Coleoptera: Coccinellidae) was collected during the study and it was found in a Bt maize suction trap. Dominant species appearing in Bt maize plots were the same as those from the non-Bt maize, which were *E. graminicolum*, *P. japonica*, *H. axyridis*, *Orius* sp., *M. tricuspidatus*, *L. sinensis*, *Chrysoperla sinica* (Tjeder) (Neuroptera: Chrysopidae), *Neoscona doenitzi* (Boes.etStr.) (Araneida: Araneidae), Aphidiidae and Aphelinidae, which accounted for 95.63% composition in Bt maize plot and 96.43% in non-Bt maize (Fig. 1D).

### Impacts of maize type and sampling time on the natural enemy diversity

Analyses of Shannon–Wiener diversity index (*H’*), Simpson’s diversity index (*D*) and Pielou’s evenness index (*J*) showed no significant differences between Bt and non Bt maize by whole plant inspections, pitfall traps and suction samplers in 2012 and 2013. The only exception was that in whole plant inspections in 2013, Pielou’s evenness index was significantly higher in Bt maize compared with non-Bt maize (*F* = 12.70, df = 1, 5, *P* = 0.016) (Table 1). During the two years, sampling time was significantly different for most of the diversity indices for each sampling method, except for Pielou’s evenness index by pitfall traps in 2013 (*F* = 3.09, df = 3, 11, *P* = 0.071), Simpson’s diversity index (*F* = 2.65, df = 3, 11, *P* = 0.101) and Pielou’s evenness index (*F* = 0.34, df = 3, 11, *P* = 0.796) by suction sampler in 2012 (Table 1). In most cases, sampling time by maize type interaction was not significant, with the exception of Simpson’s diversity index for whole plant inspections (*F* = 2.88, df = 9, 35, *P* = 0.012), and for suction sampler (*F* = 5.31, df = 3, 11, *P* = 0.017) in 2013, and Shannon–Wiener diversity index for suction sampler in 2013 (*F* = 7.95, df = 3, 11, *P* = 0.004) (Table 1).

**Figure 2:**
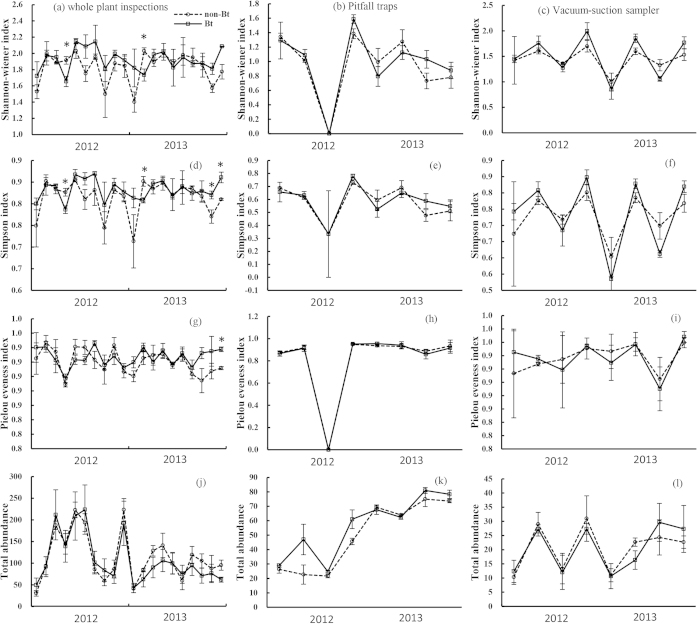
Temporal dynamics of natural enemy diversity (Shannon–Wiener diversity, Simpson’s diversity and Pielou’s evenness indices) and abundance in Bt and non-Bt maize plots in 2012 and 2013. Shannon–Wiener diversity, Simpson’s diversity and Pielou’s evenness indices by whole plant inspections (**a**,**d**,**g**,**j**), pitfall traps (**b**,**e**,**h**,**k**) and suction sampler (**c,f,i,l**); sample numbers for the two years: (20) whole plant inspections, (8) pitfall traps and (8) suction sampler. X-axis: Sampling time in 2012 and 2013 follow maize development stages: 3^rd^ (V3), 6^th^ (V6), 9^th^ (V9) 12^th^ (V12) leaf stages, tasseling (VT), silking (R1), blister (R2), milk stage (R3), dent (R5) and physiological maturity (R6). Y-axis: Mean ± SE (n = 3) of diversity indices of natural enemy community per sampling time. Asterisks (*) on sampling dates indicate significant differences based on two tailed *t*-test (α = 0.05).

We compared these significant diversity indices for sampling times with a two tailed *t*-test. All the diversity indices showed similar trends of temporal dynamics and had no consistent difference between Bt and non-Bt maize during the two years (Fig. 2a–i). For each sampling time, the results of pairwise comparison showed that for whole plant inspections, Shannon–Wiener diversity index of Bt maize was significantly different from non-Bt maize at V12 stage in 2012 (*t*-value = 3.33, *P* = 0.029), and at V6 stage (*t*-value = 3.43, *P* = 0.024) in 2013 (Fig. 2a); Simpson’s diversity index was significantly different at V12 stage in 2012 (*t*-value = 3.21, *P* = 0.033), and at V6 stage (*t*-value = 3.44, *P* = 0.026), R5 stage (*t*-value = −2.79, *P* = 0.050) and R6 stage (*t*-value = −4.04, *P* = 0.016) in 2013 (Fig. 2d); Pielou’s evenness index of Bt maize was significantly greater than non-Bt maize at R6 stage (*t*-value = −5.30, *P* = 0.013) (Fig. 2g), which may have accounted for the differences of Pielou’s evenness index between Bt and non-Bt maize in 2013. No significant difference was found in the pairwise comparisons of the diversity indices between Bt and non-Bt maize in pitfall traps and suction sampler.

### Impacts of maize type and sampling time on the natural enemy abundance

During the two years, there were no significant differences in natural enemy abundance between Bt and non-Bt maize plots. The only exception was that in pitfall traps in 2012, natural enemy abundance in Bt maize was significantly higher than non-Bt maize (*F* = 14.98, df = 1, 5, *P* = 0.012) (Table 2). Sampling time significantly affected the abundance of natural enemy collected by each sampling method, while sampling time by maize type interaction did not.

For each sampling method, natural enemy abundance showed similar trends of temporal dynamics (Fig. 2j–l). No significant difference was found in the pairwise comparisons of natural enemy abundance between Bt and non-Bt maize at each sampling time.

### Time-dependent effects of Bt maize on natural enemy community

Redundancy analysis (RDA) was performed to discern the possible relationship between natural enemy community composition and maize type, as well as sampling time. The results indicated that maize type and sampling time in total explained 12.72% of the variance of the natural enemy community data in 2012 (*F* = 4.15, *P* = 0.001; 999 Monte Carlo permutations), and 12.25% in 2013 (*F* = 3.98, *P* = 0.001; 999 Monte Carlo permutations). Among them, 11.91 and 0.81% of the variance in 2012, and 11.15 and 1.10% in 2013 were explained by the first and second axes, respectively (Table S2). When maize type and sampling time variations analyzed for contributions to natural enemy composition, maize type variance contribution (in 2012, R^2^ = 0.11, *P* = 0.028; in 2013, R^2^ = 0.14, *P* = 0.013; 999 Monte Carlo permutations) was significant, but appeared lower than that of the sampling time variance contribution (in 2012, R^2^ = 0.78, *P* = 0.001; in 2013, R^2^ = 0.68, *P* = 0.001; 999 Monte Carlo permutations) (Table S3).

**Figure 3:**
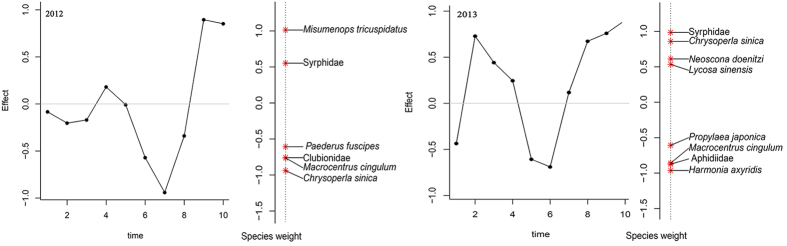
Principal response curves (PRC) representing the effects of Bt maize, in relation to non-Bt maize as a control, on the overall natural enemy communities from V3 to R6 stages in 2012 and 2013. The left Y-axis represents deviances from the control. Significant deviances based on two-tailed *t*-test of the regression coefficients (*P* < 0.05). Non-Bt maize is placed at zero. Species weights on the right with PRC curves accounting for the deviances of the PRC. Taxa with weights between −0.5 and 0.5 were removed for clarity.

Among all the canonical axes, only the first canonical axis was significant in 2012 (*F* = 7.78, *P* = 0.001; 999 Monte Carlo permutations) and 2013 (*F* = 7.25, *P* = 0.001; 999 Monte Carlo permutations), which explained 11.91 and 11.15% in 2012 and 2013 of the variation of the natural enemy community composition, respectively (Table S2). Principle response curve (PRC) analysis examined the time-dependent effects of Bt maize on the entire arthropod natural enemy community composition and considered the variations of non-Bt maize as baseline. PRC revealed that in 2012, 46.35% of the total variance of the natural enemy community data was explained by sampling time and 7.01% by maize type, as well as in 2013, 38.49% of the total variance was explained by sampling time and 10.20% by maize type. The statistical significance of the first PRC axes revealed no significant difference between the natural enemy communities in Bt and non-Bt maize plots in 2012 (*F* = 2.05, *P* = 0.969; 999 Monte Carlo permutations) (Fig. 3, Table S4), as well as in 2013 (*F* = 2.87, *P* = 0.597; 999 Monte Carlo permutations) (Fig. 3, Table S4). For simplicity Fig. 3 only summarized the species/families with species weights higher than 0.5 and lower than −0.5 in each year. In 2012, *M. tricuspidatus*, and Syrphidae in Bt maize plots were higher than those in non-Bt maize plots, whereas *C. sinica*, *Paederus Fuscipes* (Curtis) (Coleoptera: Staphylinidae), *Macrocentrus cingulum* (Brischke) (Hymenoptera: Braconidae) and Clubionidae were more likely to follow the opposite trend. Among them, *M. tricuspidatus* (*b*_*k*_ = 1.0124), and *C. sinica* (*b*_*k*_ = −0.9389) were likely to contribute most to the community response. In 2013, the abundance of Syrphidae, *C. sinica*, *N. doenitzi* and *L. sinensis* were higher in Bt maize plots than that in non-Bt maize plots, while the abundance of *P. japonica*, *H. axyridis*, Aphidiidae and *M. cingulum* were slightly lower in Bt maize plots (Table S5).

### Natural enemy assemblages in Bt and non-Bt maize plots

**Figure 4:**
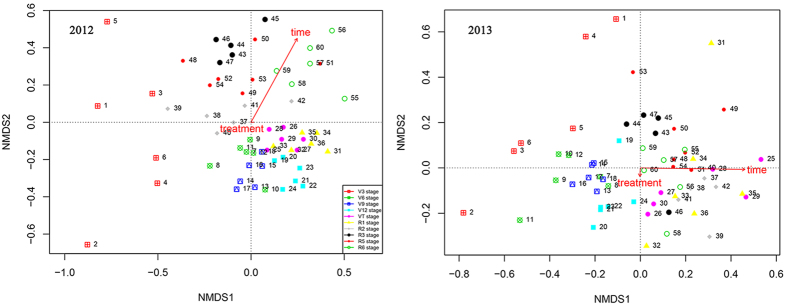
Nonmetric multidimensional scaling (nMDS) plot of natural enemy community structure from each sample by whole plant inspections in 2012 and 2013. Various shapes and associated numbers indicate sampling points analyzed in temporal order (1–3: non-Bt maize at V3 stage, 4–6: Bt maize at V3 stage, 7–9: non-Bt maize at V6 stage, 10–12: Bt maize at V6 stage, 13–15: non-Bt maize at V9 stage in 2012, 16–18: Bt maize at V9 stage, 19–21: non-Bt maize at V12 stage, 22–24: Bt maize at V12 stage, 25–27: non-Bt maize at VT stage, 28–30: Bt maize at VT stage, 31–33: non-Bt maize at R1 stage, 34–36: Bt maize at R1 stage, 37–39: non-Bt maize at R2 stage, 40–42: Bt maize at R2 stage, 43–45: non-Bt maize at R3 stage, 46–48: Bt maize at R3 stage, 49–51: non-Bt maize at R5 stage, 52–54: Bt maize at R5 stage, 55–57: non-Bt maize at R6 stage, 58–60: Bt maize at R6 stage). Different shapes are color-coded according to sampling time.

Nonmetric multidimensional scaling (nMDS) analysis (which clusters samples based on natural enemy composition) was used to visualize clusters of samples containing highly similar natural enemy composition (Fig. 4).

The nMDS plot showed that natural enemy communities in Bt and non-Bt maize were not well separated from each other for each sampling time during the two years (Fig. 4). This was supported by ANOSIM (in 2012, R^2^ = 0.00, *P* = 1.000; in 2013, R^2^ = 0.00, *P* = 0.908; 999 Monte Carlo permutations) (Fig. 4, Table S6). However, permutation tests revealed a significant correlation of nMDS structure with sampling time in 2012 (R^2^ = 0.66, *P* = 0.001; 999 Monte Carlo permutations) and 2013 (R^2^ = 0.48, *P* = 0.001; 999 Monte Carlo permutations). According to a multivariate permutation test on measuring the effects of sampling time and maize type on Bray–Curtis distance, we found that sampling time shaped Bray–Curtis distance by 61.24% in 2012 (*F* = 8.69, *P* = 0.001; 999 Monte Carlo permutations) and 53.33% in 2013 (*F* = 6.33, *P* < 0.001; 999 Monte Carlo permutations), but maize type had no effect on Bray–Curtis distance (in 2012: *F* = 0.48, *P* = 0.809; in 2013: *F* = 0.82, *P* = 0.538; 999 Monte Carlo permutations) (Table 3).

## Discussion

Most studies that have considered possible effects of Bt maize on non-target arthropods, including natural enemies, have focused on maize expressing Cry3Bb1^36^ or Cry1Ac^37^ insecticidal proteins. Only one study reported on possible effects of transgenic *cry1Ie* maize on the entire non-target arthropod biodiversity in the field^35^, and no effects were found. Because of the ecological importance of natural enemies, the natural enemy subset of this study that included whole plant inspections, pitfall traps and suction sampler was evaluated in more detail.

Methods for analyzing the effects of environmental factors on species and ecological communities are generally fallen into two categories: (i) those that emphasize on the distributions of individual species, and (ii) those that emphasize on differences in the community composition^38^. Since one single method is not available to completely evaluate the natural enemy community, a combination of methods was necessarily performed to obtain a more comprehensive view of the natural enemy community in Bt and non-Bt maize plots. Therefore, diversity indices (diversity: Shannon-Weiner’s, and Simpson’s; evenness: Pielou’s), abundance index, redundancy analyses (RDA), principal response curves (PRC) and nonmetric multidimensional scaling (nMDS) were used within this study.

The two diversity indices (Shannon-Weiner’s, and Simpson’s) and an evenness index (Pielou’s) are useful indicators for measuring the disturbance of natural enemy communities^37^. Significant effect of maize type was observed only for Pielou’s evenness index with whole plant inspections in 2013, where Bt maize was higher than that of non-Bt maize (Table 1). These results overall suggested that Cry1Ie Bt maize had little to no impact on natural enemy community biodiversity. However, when these indices were used to evaluate different stages of maize development, some differences were detected for whole plant inspections, but these differences showed no clear pattern (Fig. 2). Significant differences between Bt and non-Bt maize plots were observed at V12 stage in 2012 and V6 stage in 2013 for Shannon-winner index, at V12 stage in 2012 and V6, R5 and R6 stages in 2013 for Simpson’s diversity index, and R6 stage in 2013 for Pielou’s evenness index by whole plant inspections (Fig. 2a,d,g). Interestingly, in 2013 indices were higher in Bt maize plots than non-Bt maize plots for Simpson’s diversity index during R5 and R6 stages, and Pielou’s evenness index during R6 stage (Fig. 2d,g). Overall, these results suggested that the diversity indices between Bt and non-Bt maize had relatively minor differences and these differences showed no consistent pattern. In some cases, the Shannon-Weiner’s and Simpson’s diversity indices may have been influenced by rarely occurring species, which occurred more often in non-Bt maize compared to Bt maize, e.g., *M. cingulum*. No significant differences existed in natural enemy abundance between Bt and non-Bt maize except for pitfall traps in 2012, where we found fewer natural enemies in non-Bt maize plots compared with Bt maize plots (Table 2). These findings were consistent with a previous 2-yr monitoring survey report that Bt maize expressing Cry1Ac proteins did not affect the natural enemy community^37^. Similarly, a 3-yr study reported that no detrimental effect of farm-scale Bt maize was observed on abundance of predatory arthropods in Spain^21^.

PRC is a multivariate technique used to assess the structure of arthropod and soil nematode community in Bt and non-Bt crop^37^,^39^. Two fundamental questions for Bt maize environmental risk assessment for natural enemies were answered by using PRC models for whole plant inspections in 2012 and 2013. First, does Bt maize alter the natural enemy composition in a series of repeated observation? Results clearly suggested there were no consistent differences over time as there were no significant effects of Bt maize on natural enemy population distribution over time when compared to non-Bt maize in 2012 and 2013 (Fig. 3). Second, how do individual natural enemy species respond to Bt and non-Bt maize (vertical line of right side of line charts; Fig. 3)? Among the 14 species/families monitored in 2012, *C. sinica*, *P. fuscipes*, *M. cingulum* and Clubionidae were more abundant in non-Bt maize plots, but *M. tricuspidatus* and Syrphidae were more abundant in Bt maize. Among 22 species/families in 2013, *P. japonica*, *H.axyridis*, Aphidiidae and *M. cingulum* were more abundant in non-Bt maize, but Syrphidae, *C. sinica*, *N. doenitzi*, and *L. sinensis* were more abundant in Bt maize. The only consistent results were that Syrphidae was higher in Bt maize and *M. cingulum*, a specialist larval parasitoid, was higher in non-Bt maize. The latter is probably attributed to fewer Asian corn borers, *O. furnacalis*, in Bt maize.

The RDA allowed one to assess the relative contributions of maize type, sampling time and unknown factors with the abundances and species of natural enemies. Maize type and sampling time explained 12.72 and 12.25% of the variance in the natural enemy communities in 2012 and 2013, respectively. More than 80% of the variance was caused by other factors. Many factors may explain the differences besides genetic modification: (1) plot size and isolation among them determine the abundance of aboveground arthropods^40^; (2) fewer target insects in Bt crop plots cause less damage in the plots, so the effects of herbivore-induced volatile in plants to natural enemies may be weak^41^,^42^; (3) effects of the reduced number of target pests on natural enemy community abundance, and (4) transgenic crops with new genes could alter the physiological parameters^43^ (i.e., lower N accumulation or differential plant development rate^44^). A decline in target hosts is a likely mechanism to explain the reduction of natural enemies^45^. For example, fewer maize borers probably contributed to fewer *M. cingulum*, an *Ostrinia* larval parasitoid, in Bt maize plots.

The Bray-Curtis distances for 2012 and 2013 showed that sampling time was a much more important factor than maize type in explaining natural enemy variability (Fig. 4). NMDS is less sensitive than the diversity indices to rare species occurrence in samples so may be more appropriate for comparing community compositions of Bt and non-Bt maize^46^.

Natural enemies contribute to the control of pest populations^47^, thus natural enemy biodiversity is linked to biological control of insect pests^48^. Transgenic crops can reduce pesticide applications^18^. If the abundance and diversity of natural enemies is maintained in transgenic crops because fewer insecticides are used, secondary pests are less likely to outbreak. Ultimately, Bt maize could foster durable integrated pest management^26^,^49^.

Assessment of possible impacts of Bt maize on natural enemy community and biodiversity is necessary for a pre-release environmental risk assessment^50^ and post-release monitoring^51^. Taken as a whole, our study confirmed the results of similar field trials of Bt maize expressing Cry1Ac insecticidal proteins^37^ demonstrating that Cry proteins expression in maize did not affect natural enemies populations (predators, parasitoids). Laboratory studies further confirm that direct feeding on Bt plant material poses a negligible risk for predators^22^,^52^. Field conditions are complex, and many factors cannot be controlled, weather, different prey types, food shortage, prey choice and competition between natural enemies and all these factors may influence the final results.

This study confirmed that despite relatively minor differences in natural enemy communities between Bt and non-Bt maize, transgenic *cry1Ie* maize had little effect on natural enemy community biodiversity. This 2-yr field assessment provided an assessment of possible ecological effects of Bt maize on natural enemy biodiversity. The indirect effects of Bt maize on natural enemy, such as the attraction of herbivore-induced volatile emission in maize on natural enemy^41^, or the abundance of food resources for natural enemies, were not separated from the direct effects. In addition, which natural enemies could ingest Bt insecticidal proteins when living/feeding on the hosts/prey were not determined. Such factors could be deciphered with additional long-term studies.

## Methods

### Ethics statement

For natural enemies sampling in maize field, no specific permits were required.

None of the species used in this study are endangered or protected.

### Experimental design

**Figure 5:**
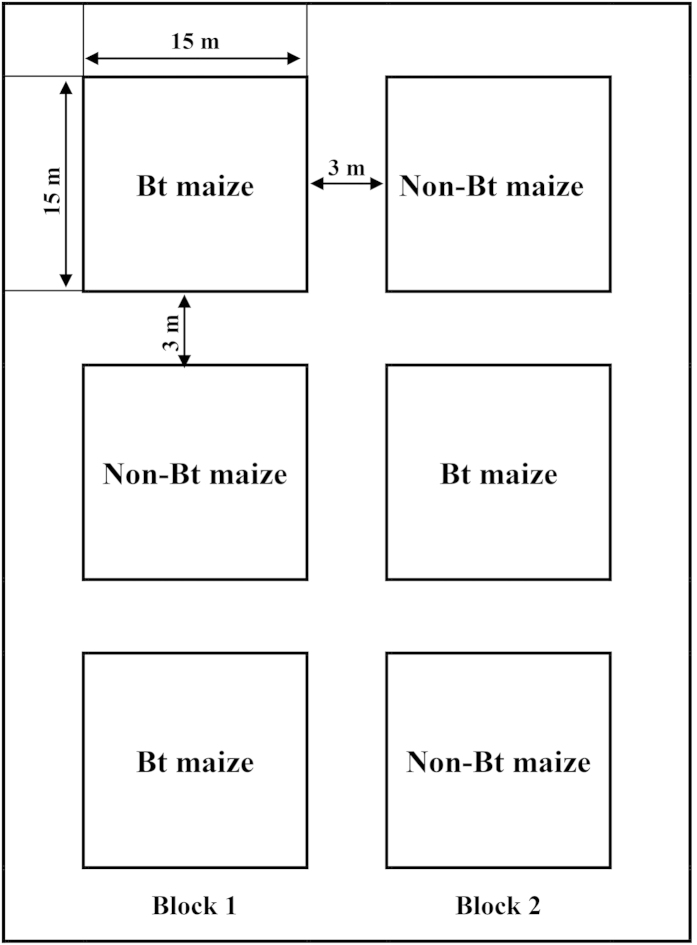
Distribution of plots for Bt and non-Bt maize in the fields in 2012 and 2013.

The study was conducted in 2012 and 2013 at the Langfang Experiment Station of Institute of Plant Protection, Chinese Academy of Agricultural Sciences, Hebei province (39°30′N, 116°36E). The seeds of Bt maize (Event IE09S034) and its near-isogenic non-Bt counterpart (Zong 31) maize used in this experiment were provided by Institute of Crop Sciences, Chinese Academy of Agricultural Sciences. Maize was planted on 8 May 2012, and 29 May 2013. A randomized block design involving two maize types (Bt and non-Bt maize) was established with three replications (Fig. 5). Each plot was 15 by 15 m and consisted of 25 rows with 60-cm space among them and 35-cm space among individual plants. Plots were separated by three meter bare borders. Plots were cultivated using standardized agricultural management practices but no insecticides were applied during the study.

### Sample collection

Whole plant inspections were carried out periodically for all the main growing stages of maize: 3^rd^ (V3), 6^th^ (V6), 9^th^ (V9) 12^th^ (V12) leaf stages, tasseling (VT), silking (R1), blister (R2), milk stage (R3), dent (R5) and physiological maturity (R6) in 2012 and 2013. In each Bt maize and non-Bt maize plots, twenty plants were randomly selected along two corner-to-corner diagonals (X shaped) (100 total plants per plot sampled each year). The numbers of visible natural enemies on stalks, leave, sheaths, tassels, husks and ears were quickly counted. If a species could not be identified, it was kept within a 5-ml plastic tube with 75% alcohol for later identification.

Pitfall traps and suction sampler were also used to collect natural enemies at V6, V9, R1 and R2 stages of maize development. Five sites were chosen in each plot, one site was in the plot center and the other four were in the middle of lines that connected the plot center with plot corners. Three pitfall traps spaced 0.5m apart in a line were established at each of the five locations in a plot. For each trap a plastic outer cup (15 cm in diameter × 10 cm depth) was buried in the ground with the upper rim of the cup level with the ground. Then one collection cup (5 cm in bottom diameter × 8 cm depth) with 75% ethanol, sugar, vinegar and water was put into each outer cup. Traps were exposed for about 24 h, after which they were collected and trapped natural enemies were stored in 75% ethanol until they were identified to species and counted. A Univac suction sampler (Burkard Manufacturing Co. Ltd, UK) was used to collect natural enemies, especially for the small individuals not easy to observe from 10 plants for each of the trapping locations. All the collected arthropods again, if possible, were identified to species.

### Statistical analysis

To analyze the natural enemy abundance and diversity, the number of species (species richness) and the relative abundances of individuals within each species (species abundance) per plot at each sampling time were calculated. The total number of individuals caught per plot at each sampling time was used as an index of relative abundance^53^. The diversity of natural enemy community at each plots and sampling time was evaluated by Shannon-Weiner’s, Pielou’s and Simpson’s index. Shannon-Weaver diversity index (*H’*) was then calculated as follows:


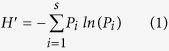


where *P*_*i*_is the proportion of individuals belongs to the *i*^th^ taxon in the total number of individuals^54^.

Pielou’s evenness index (*J*) was calculated as follows:


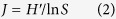


where *S* is the total number of genera, *H′* is Shannon-Weaver diversity index^55^.

Simpson’s diversity index (*D*) was calculated as follows:


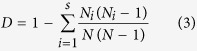


*N*_*i*_ = the number of individuals in the *i*^th^ taxon; and *N* = the total number of individuals^56^.

Those three diversity indices in whole plant inspections, pitfall traps and suction sampler in 2012 and 2013 were calculated using the ‘diversity’ function of the vegan package^57^ in R. Total number of natural enemies were log(x + 1) transformed before calculating diversity indices.

Unequally spaced repeated-measures ANOVA (‘proc mixed’ procedure in SAS)^58^ was used to test the effects of maize type and sampling time on natural enemy abundance, Shannon-Weiner’s, Simpson’s and Pielou’s index. Maize type and sampling time were fixed factors, and block was a random factor. When a significant effect of sampling time was observed, comparison of mean values of abundance, *H*′, *J* and *D* on each sampling time was conducted with a two-tailed *t*-test to detect significant differences between Bt and non-Bt maize plots.

One canonical analysis method (redundancy analysis, RDA), and its special type (principal response curve, PRC), and one indirect ordination method (nonmetric multidimensional scaling, nMDS) were used to compare differences in the composition of natural enemy communities and identify environmental controls of community composition statistically^38^. Here we only analyze the data collected by whole plant inspections in 2012 and 2013 due to the higher abundance of natural enemies.

RDA was performed to investigate any linear relationships between the factors (maize type and sampling time) and the variation in natural enemy community composition. DCA was initially used to explore the range of variation within the natural enemy community composition^59^. If the longest gradient length was less than 3, the species were responding linearly to the gradients of explanatory variables, and therefore, linear response models were appropriate^60^. In our present study, the longest gradient length of DCA in 2012 and 2013 were short (1.74 and 1.52 SD, respectively), so RDA was chosen.

RDA is a constrained ordination technique in which the main axes are constrained to be linear combinations of the environmental variables^60^. Here we used RDA directly displayed the variation of natural enemy species as much as they can be explained by maize type and sampling time^61^ using the ‘rda’ function of the vegan package^57^ in R. The proportion of variance explained by sampling time and maize type was quantified using R^2^ (adjusted)^62^. The total abundances of natural enemy were log(x + 1) transformed prior to analysis to stabilized variance and reduce the influence of dominant taxa on the ordination^63^. The level of significance of the canonical axes of RDA axes was tested by Monte-Carlo permutation test in R and yielded only one significant axis in each year. Thus the species–explanatory variables correlation along the first axis of RDA was used to set up the PRC.

PRC was used to visualize temporal changes in natural enemy composition caused by Bt maize as compared with non-Bt maize and also to quantify the contribution of each species to separate Bt maize from non-Bt maize (*prc* function, Vegan package in R). PRC is a special type of RDA for multivariate responses in a design with a series of repeated observation^64^. In the PRC analysis, principal component in species composition is plotted through time for explaining compositional deviations from the control represented as a horizontal line^65^. This is achieved by taking the non-Bt maize as the reference to Bt maize and by defining “time” as the horizontal axis of the diagram. The species weights (*b*_*k*_) represent the highly affinity of the treatment, and indicate the direction of the changes in abundance^66^. Species weights between +0.5 and −0.5 were not displayed for their weak or unrelated responses to the PRC^67^. Monte Carlo permutations test was performed to test the significance between Bt maize and non-Bt maize for the axis of interest (in our case is the first axis), and the critical probability level for detecting significance between Bt and non-Bt was set at α = 0.01^67^.

**Figure 6:**
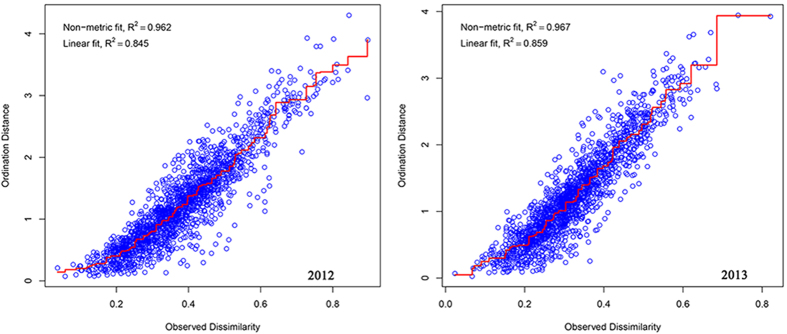
Nonmetric Multidimensional Scaling (nMDS) Shepard plot of natural enemy community by whole plant inspections in 2012 and 2013.

The similarities of natural enemy community composition across maize type and sampling time were visualized using nMDS based on Bray–Curtis dissimilarity matrix^68^. A stress function ranged from 0 to 1 was used to assess the goodness of fit between the ordination and the original data of natural enemies (*isoMDS* function, Vegan package in R). The stress values were all below 0.2 (2012 was 0.1950, and 2013 was 0.1817), which suggested that the ordination was accurately represented the dissimilarity between samples^69^. Shepard diagram (*stress plot* function, Vegan Package in R) of non-metric fit illustrated that observed dissimilarities and the ordination distances were high correlated (non-metric fit was 0.962 in 2012, and 0.967 in 2013, respectively) (Fig. 6). In our study, the natural enemies surveyed in Bt and non-Bt maize by whole plant inspection in 2012 and 2013 distributed into 60 samples (10 sampling time × 2 maize types × 3 replications). Data were log (x + 1) transformed and normalized before Bray–Curtis dissimilarity were computed.

NMDS produced a two-dimensional graphical representation of the pairwise dissimilarity between natural enemy communities in each sample using the packages Vegan and Mass of R version 3.0.3^56^. Distances between each plot were estimated by using Bray-Curtis dissimilarity index^70^, which is one of the most robust statistics for multivariate ecological analysis of community composition among samples and slightly affected by the presence of rare species^46^.

NMDS also allowed us to determine which measured factors (maize type and sampling time) were significantly correlated with the nMDS ordination of natural enemy communities by returning squared correlation coefficients (*envfit* function, Vegan package in R)^57^. Fitted vectors were plotted onto the nMDS ordination of natural enemy community structures in Bt and non-Bt maize plots, and significance of fitted vectors were assessed by a permutation test^57^.

We likewise used Bray–Curtis dissimilarity index to determine whether changes in natural enemy community composition between samples were related to changes in maize type and sampling time with ‘Adonis’ function in the vegan package^57^ of R.

Statistical analyses were performed in the R software environment (v.3.0.3; R Development Core Team)^71^ and SAS statistics package version 9.2^72^.

## Additional Information

**How to cite this article**: Guo, J. *et al.* Field trials to evaluate the effects of transgenic *cry1Ie* maize on the community characteristics of arthropod natural enemies. *Sci. Rep.* **6**, 22102; doi: 10.1038/srep22102 (2016).

## Supplementary Material

Supplementary Information

## Figures and Tables

**Table 1:** Effects of maize type and sampling time on natural enemy diversity and evenness in 2012 and 2013.

	Diversity indices	Year	Non-Bt maize	Bt maize	Maize type		Sampling time		Maize type × Sampling time	
					*F*	*P*	*F*	*P*	*F*	*P*
Whole plant inspections	Shannon’s diversity index (*H’*)	2012	1.83 ± 0.06	1.94 ± 0.05	4.01	0.1017	6.16	**<0.0001**	1.51	0.1830
		2013	1.83 ± 0.06	1.90 ± 0.03	1.49	0.2771	3.51	**0.0035**	2.26	0.0176
	Simpson’s diversity index (*D*)	2012	0.82 ± 0.01	0.83 ± 0.01	2.69	0.1621	7.68	**<0.0001**	1.31	0.2694
		2013	0.81 ± 0.01	0.83 ± 0.01	2.87	0.1511	4.40	**0.0007**	2.88	**0.0118**
	Pielou’s evenness index (*J*)	2012	0.93 ± 0.01	0.92 ± 0.00	0.04	0.8553	5.21	**0.0002**	1.19	0.3344
		2013	0.91 ± 0.00 b	0.93 ± 0.00 a	12.70	**0.0161**	2.90	**0.0114**	1.58	0.1601
Pitfall traps	Shannon’s diversity index (*H’*)	2012	0.93 ± 0.32	0.99 ± 0.35	0.42	0.5450	246.77	**<0.0001**	1.48	0.2742
		2013	0.94 ± 0.12	0.96 ± 0.07	0.02	0.9046	5.21	**0.0176**	2.17	0.1493
	Simpson’s diversity index (*D*)	2012	0.59 ± 0.09	0.60 ± 0.09	0.01	0.9333	3.66	**0.0475**	0.03	0.9930
		2013	0.57 ± 0.05	0.58 ± 0.03	0.06	0.8237	4.46	**0.0279**	1.48	0.2725
	Pielou’s evenness index (*J*)	2012	0.69 ± 0.23	0.68 ± 0.23	0.15	0.7128	2212.37	**<0.0001**	0.12	0.9468
		2013	0.92 ± 0.01	0.91 ± 0.02	0.07	0.7970	3.09	0.0716	0.48	0.7026
Suction sampler	Shannon’s diversity index (*H’*)	2012	1.52 ± 0.08	1.62 ± 0.16	0.94	0.3770	3.95	**0.0390**	0.48	0.7028
		2013	1.36 ± 0.14	1.38 ± 0.25	0.04	0.8558	57.73	**<0.0001**	7.95	**0.0043**
	Simpson’s diversity index (*D*)	2012	0.74 ± 0.03	0.77 ± 0.04	0.70	0.4424	2.65	0.1006	0.36	0.7831
		2013	0.71 ± 0.04	0.70 ± 0.07	0.23	0.6494	47.48	**<0.0001**	5.31	**0.0166**
	Pielou’s evenness index (*J*)	2012	0.93 ± 0.01	0.94 ± 0.01	0.11	0.7560	0.34	0.7959	0.22	0.8787
		2013	0.94 ± 0.02	0.94 ± 0.01	0.38	0.5642	3.75	**0.0447**	0.17	0.9173

All data were analyzed using a 2-way unequally spaced repeated-measure ANOVA. Values highlighted in bold are statistically significant (*P* < 0.05). Values for Bt and non-Bt maize followed by the different lowercase letters within a row are significantly different using *t*-tests (P < 0.05).

**Table 2:** Effects of maize type and sampling time on natural enemy abundance in 2012 and 2013.

Total abundance	Year	Non-Bt maize	Bt maize	Maize type		Sampling time		Maize type × Sampling time	
				*F*	*P*	*F*	*P*	*F*	*P*
Whole plant inspections	2012	132.80 ± 22.34	131.17 ± 20.98	0.64	0.4602	48.58	**<0.0001**	1.11	0.3816
	2013	96.2 ± 9.60	77.97 ± 6.23	4.59	0.0850	7.50	**<0.0001**	1.28	0.2817
Pitfall traps	2012	29.08 ± 5.62 b	40.42 ± 8.39 a	14.98	**0.0118**	19.46	**<0.0001**	2.97	0.0785
	2013	70.5 ± 2.48	72.33 ± 4.41	1.75	0.2437	21.13	**<0.0001**	1.94	0.1813
Suction sampler	2012	20.92 ± 5.30	19.75 ± 4.38	0.30	0.6090	8.62	**0.0031**	0.20	0.8943
	2013	20.17 ± 3.08	21 ± 4.51	0.13	0.7218	8.00	**0.0042**	1.01	0.4276

All data were analyzed using a 2-way unequally spaced repeated-measure ANOVA. Values highlighted in bold are statistically significant (*P* < 0.05). Values for Bt and non-Bt maize followed by the different lowercase letters within a row are significantly different using t-tests (*P* < 0.05).

**Table 3:** Effects of maize type and sampling time on beta diversity (Bray–Curtis distance) of natural enemy community.

Year	Adonis on Bray–Curtis distances	df	Sums of Squares	*F*-statistics	R^2^	*P*-value
2012	Maize type	1	0.018	0.48	0.00	0.809
	Sampling time	9	2.920	8.69	0.61	0.001 ***
	Residuals	49	1.828	–	0.38	–
	Total	59	4.767	–	–	–
2013	Maize type	1	0.027	0.82	0.00	0.538
	Sampling time	9	1.846	6.33	0.53	0.001***
	Residuals	49	1.589	–	0.46	–
	Total	59	3.461	–	–	–

^***^P < 0.001.
