# Supplementary material for: Field trials to evaluate the effects of transgenic cry1Ie maize on the community characteristics of arthropod natural enemies
Source: Sci Rep. 2016 Feb 26;6:22102. doi: 10.1038/srep22102 (PMC4768170; doi:10.1038/srep22102)
Supplement: Supplementary Information [file srep22102-s1.pdf]

Supplementary Information

Field trials to evaluate the effects of transgenic *cry1Ie* maize on the community characteristics of arthropod natural enemies

Jingfei Guo[1], Kanglai He[1], Richard L Hellmich[2], Shuxiong Bai[1], Tiantao Zhang[1], Yunjun Liu[3], Tofael Ahmed[1,4], Zhenying Wang[1]*

[1]State Key Laboratory for Biology of Plant Diseases and Insect Pests, MOA – CABI Joint Laboratory for Bio-safety, Institute of Plant Protection, Chinese Academy of Agricultural Sciences, Beijing 100193, China.

[2]USDA-ARS, Corn Insects and Crop Genetics Research Unit, Ames, IA 50011, USA.

[3]Institute of Crop Sciences, Chinese Academy of Agricultural Sciences, Beijing 100081, China.

[4]Entomology Division, Bangladesh Sugarcane Research Institute, Ishurdi, Pabna,

*Corresponding author Email: zywang@ippcaas.cn.

Supplementary Table S1 Abundance and percentage of natural enemies collected in Bt and non-Bt maize plots in 2012 and 2013 by three methods and methods combined

	Family/species	Whole plant inspections				Pitfall traps				Suction sampler				Methods combined			
		Bt maize		Non-Bt maize		Bt maize		Non-Bt maize		Bt maize		Non-Bt maize		Bt maize		Non-Bt maize	
		No.	Ratio (%)	No.	Ratio (%)	No.	Ratio (%)	No.	Ratio (%)	No.	Ratio (%)	No.	Ratio (%)	No.	Ratio (%)	No.	Ratio (%)
1	*Propylea japonica*	1247	19.11	1535	22.59	1	0.30	0	0.00	72	15.00	65	17.47	1320	17.97	1600	21.08
2	*Harmonia axyridis*	1363	20.88	1334	19.63	1	0.30	1	0.24	114	23.75	48	12.90	1478	20.13	1383	18.22
3	*Misumenops tricuspidatus*	361	5.53	284	4.18	0	0.00	0	0.00	7	1.46	10	2.69	368	5.01	294	3.87
4	*Erigonidium graminicolum*	1718	26.32	2011	29.60	15	4.45	25	5.91	87	18.13	87	23.39	1820	24.78	2123	27.97
5	*Orius* sp	1203	18.43	1080	15.89	18	5.34	7	1.65	30	6.25	48	12.90	1251	17.03	1135	14.95
6	*Macrocentrus cingulum*	57	0.87	17	0.25	0	0.00	0	0.00	4	0.83	0	0.00	61	0.83	17	0.22
7	Aphidiidae	25	0.38	23	0.34	0	0.00	0	0.00	3	0.63	4	1.08	28	0.38	27	0.36
8	Aphelinidae	71	1.09	68	1.00	1	0.30	2	0.47	6	1.25	4	1.08	78	1.06	74	0.97
9	Syrphidae	37	0.57	25	0.37	0	0.00	0	0.00	2	0.42	6	1.61	39	0.53	31	0.41
10	*Chrysoperla sinica*	176	2.70	179	2.63	0	0.00	0	0.00	27	5.63	15	4.03	203	2.76	194	2.56
11	*Paederus fuscipes*	146	2.24	123	1.81	2	0.59	2	0.47	21	4.38	8	2.15	169	2.30	133	1.75

12	Clubionidae	28	0.43	33	0.49	7	2.08	13	3.07	12	2.50	10	2.69	47	0.64	56	0.74
13	*Neoscona doenitzi*	21	0.32	28	0.41	115	34.12	96	22.70	1	0.21	1	0.27	137	1.87	125	1.65
14	*Lycosa sinensis*	36	0.55	21	0.31	120	35.61	207	48.94	43	8.96	30	8.06	199	2.71	258	3.40
15	*Lydella grisescens*	3	0.05	6	0.09	29	8.61	31	7.33	4	0.83	3	0.81	36	0.49	40	0.53
16	Asilidae	9	0.14	7	0.10	0	0.00	0	0.00	4	0.83	1	0.27	13	0.18	8	0.11
17	*Aphidoletes aphidimyza*	1	0.02	0	0.00	0	0.00	0	0.00	21	4.38	3	0.81	22	0.30	3	0.04
18	Reduviidae	1	0.02	3	0.04	2	0.59	2	0.47	1	0.21	1	0.27	4	0.05	6	0.08
19	*Nebria livida*	0	0.00	3	0.04	19	5.64	16	3.78	2	0.42	6	1.61	21	0.29	25	0.33
20	*Geocoris pallidipennis*	0	0.00	0	0.00	0	0.00	0	0.00	6	1.25	3	0.81	6	0.08	3	0.04
21	Cicindelidae	7	0.11	3	0.04	0	0.00	0	0.00	3	0.63	1	0.27	10	0.14	4	0.05
22	Ichneumonidae	1	0.02	1	0.01	7	2.08	16	3.78	0	0.00	3	0.81	8	0.11	20	0.26
23	*Trichogramma ostriniae*	3	0.05	6	0.09	0	0.00	5	1.18	5	1.04	11	2.96	8	0.11	22	0.29
24	*Polistes okinawensis*	13	0.20	5	0.07	0	0.00	0	0.00	1	0.21	1	0.27	14	0.19	6	0.08
25	*Coccinella septempunctata*	0	0.00	0	0.00	0	0.00	0	0.00	1	0.21	0	0.00	1	0.01	0	0.00
26	Nabidae	0	0.00	0	0.00	0	0.00	0	0.00	1	0.21	1	0.27	1	0.01	1	0.01
27	*Adonia variegata*	0	0.00	0	0.00	0	0.00	0	0.00	2	0.42	2	0.54	2	0.03	2	0.03

Total (Abundance and No. family/species)	6527(22)	6795(22)	337(13)	423(13)	480(26)	372(25)	7344(27)	7590(26)

Supplementary Table S2 Summary of the redundancy analysis of natural enemy communities by whole plant inspections in 2012 and 2013

Year	Axes	Eigenvalue	Proportion	Permutation tests for axes			
				Df	Variance	*F*	Pr (>*F*)
2012	RDA1	1.6678	11.91%	1	1.6678	7.7798	**0.001*** **
	RDA2	0.1131	0.81%	1	0.1131	0.5275	0.899
	Accumulated constrained eigenvalues	1.7809	12.72%				
	Permutation test for all constrained eigenvalues (999 Monte Carlo permutations)						
	Pseudo-*F:*	4.1537					
	Significance:	**0.001*** **					
2013	RDA1	1.5616	11.15 %	1	1.5616	7.2452	**0.001*** **
	RDA2	0.1533	1.10%	1	0.1533	0.7111	0.726
	Accumulated constrained eigenvalues	1.7149	12.25%				
	Permutation test for all constrained eigenvalues (999 Monte Carlo permutations)						
	Pseudo-*F*:	3.9781					
	Significance:	**0.001*** **					

***, $P < 0.001$

Supplementary Table S3 Significance of maize type (Bt maize expressing Cry1Ie proteins, non-Bt maize Zong 31) and sampling time on distribution of natural enemies (RDA structure) by whole plant inspections in 2012 and 2013

Correlation with RDA structure	2012		2013	
	R^2	*P*-value	R^2	*P*-value
Maize type	0.1146	**0.028***	0.1369	**0.014***
Sampling time	0.7765	**0.001*** **	0.6796	**0.001*** **

*, $P < 0.05$; ***, $P < 0.001$

Supplementary Table S4 Percentages of the total variance that can be attributed to sampling time and maize type in the first principal response curves (PRC) and results of significance tests (Monte Carlo permutation tests, 999) of the first PRC for natural enemy community by whole plant inspections in 2012 and 2013

	2012		2013	
	Maize type	Sampling time	Maize type	Sampling time
% Variance	7.01	46.35	10.20	38.49
Pseudo-*F*	2.0493		2.8681	
Significance	0.969		0.597	

Supplementary Table S5 The species weights (b_k) in principle response curves (PRC) by visual observation in 2012 and 2013. The species weights above +0.5 and below -0.5 were highlighted in bold. The species that their total number in each year less than five were not analyzed by PRC

Family/species	2012	2013
Propylaea japonica	0.09276	**-0.6081**
Harmonia axyridis	-0.27855	**-0.9632**
Misumenops tricuspidatus	**1.01241**	0.1609
Erigonidium graminicolum	-0.21081	-0.3045
Orius.sp.	-0.38726	0.1397
Macrocentrus cingulum	**-0.76340**	**-0.8590**
Aphidiidae	0.36586	**-0.8743**
Aphelinidae	-0.47069	0.2582
Syrphidae	**0.55212**	**0.9853**
Chrysoperla sinica	**-0.93892**	**0.8585**
Paederus fuscipes	**-0.60754**	0.3388
Clubionidae	**-0.75933**	-0.1601
Neoscona doenitzi	-0.24867	**0.6113**
Lycosa sinensis	0.07137	**0.5346**

Supplementary Table S6 Effects of maize type (Bt-maize expressing Cry1Ie proteins, non-Bt maize, Zong 31) and sampling time on natural enemy community structure (nMDS structure) by whole plant inspections in 2012 and 2013

Correlation with nMDS structure	2012		2013	
	R^2	*P*-value	R^2	*P*-value
Maize type	0.0001	1.000	0.0033	0.908
Sampling time	0.6648	**0.001*****	0.4750	**0.001*****

***, $P < 0.001$
